# Physiological Ischemic Training Promotes Brain Collateral Formation and Improves Functions in Patients with Acute Cerebral Infarction

**DOI:** 10.3389/fneur.2016.00235

**Published:** 2016-12-22

**Authors:** Xiaoyue Zhen, Yu Zheng, Xunning Hong, Yan Chen, Ping Gu, Jinrong Tang, Hong Cheng, Ti-Fei Yuan, Xiao Lu

**Affiliations:** ^1^The First Affiliated Hospital of Nanjing Medical University, Nanjing, China; ^2^Beijing Rehabilitation Hospital of Capital Medical University, Beijing, China; ^3^Department of Rehabilitation Medicine, West China Hospital, Sichuan University, Chengdu, China; ^4^Institute for Disaster Management and Reconstruction, Sichuan University – Hong Kong Polytechnic University, Chengdu, China; ^5^Interdisciplinary Division of Biomedical Engineering, Hong Kong Polytechnic University, King’s Park, Hong Kong; ^6^School of Psychology, Nanjing Normal University, Nanjing, China

**Keywords:** physiological ischemic training, vascular endothelial growth factor, endothelial progenitor cells, cerebral blood flow, stroke

## Abstract

**Objectives:**

To observe the effectiveness and mechanisms of physiological ischemic training (PIT) on brain cerebral collateral formation and functional recovery in patients with acute cerebral infarction.

**Methods:**

20 eligible patients with acute cerebral infarction were randomly assigned to either PIT group (*n* = 10) or Control group (*n* = 10). Both groups received 4 weeks of routine rehabilitation therapy, while an additional session of PIT, which consisted of 10 times of maximal voluntary isometric handgrip for 1 min followed by 1 min rest, was prescribed for patients in the PIT groups. Each patient was trained with four sections a day and 5 days a week for 4 weeks. The Fugl–Meyer Assessment (FMA), the Modified Barthel Index (MBI), and the short-form 36-item health survey questionnaire (SF-36) were applied for the evaluation of motor impairment, activity of daily living, and quality of life at the baseline and endpoint. MRI was applied to detect the collateral formation in the brain. The concentration of vascular endothelial growth factor (VEGF) and endothelial progenitor cells (EPCs) number in plasma were also tested at the endpoint.

**Results:**

Demographic data were consistent between experimental groups. At the endpoint, the scores of the FMA, MBI, and SF-36 were significantly higher than that at baseline. As compared to the Control group, the score of FMA and SF-36 in PIT group was significantly higher, while no significant difference was detected between groups in terms of MBI. Both groups had significantly higher cerebral blood flow (CBF) level at endpoint as compared to that at baseline. Moreover, the CBF level was even higher in the PIT group as compared to that in the Control group after 4 weeks of training. The same situations were also found in the plasma VEGF and EPCs assessment. In addition, positive correlations were found between FMA score and CBF level (*r* = 0.686, *p* < 0.01), CBF level and VEGF concentration (*r* = 0.675, *p* < 0.01), and VEGF concentration and EPC number (*r* = 0.722, *p* < 0.01).

**Conclusion:**

PIT may be effective in increasing the expression of VEGF and recruitment of EPCs and in turn promote the formation of brain collateral circulation. The positive correlations may demonstrate a potential association between biological and functional parameters, and PIT may be able to improve the motor function, activity of daily living, and quality of life in patients with stroke.

## Introduction

Stroke is one of the leading causes of disability with high morbidity and mortality. The incidence of stroke per 100,000 person-year is 376 for men and 264 for women in China (1). Of all the patients, 87% are ischemic stroke (2). For decades, physicians and scientists have focused on the development of neuroprotection, such as anti-apoptosis, anti-inflammatory, anti-oxidant, or calcium channel blockers. However, all of these treatment have their specific limitations in early application; for example, RT-PA, due to its application is limited by the thrombolytic time window, can be only applied to a few patients. Therefore, there are still challenges in improving the outcomes of stroke patients.

The proliferation of endothelial cells and growth of blood vessels, primarily in the penumbra, enhances the oxygen and nutrient supply to the ischemia-affected tissue and facilitates neurogenesis and synaptogenesis, which in turn lead to improved functional recovery (3). Animal studies showed that transient ischemic stimulation could decrease the cerebral infarction size (4). One of the clinical studies reported that transient ischemic attack before ischemic stroke could alleviate neurological deficits and improve the outcomes of acute stroke patients (5). However, intended ischemic stimulation *in situ* is not possible to perform in human due to the safety issues.

Our previous studies showed that ischemic electrical stimulation on normal limbs can facilitate collateral formation on local ischemic limbs and remote myocardium of rabbit (6, 7); similar outcomes were also reported with isometric training (8), which was named as physiological ischemic training (PIT). It is featured by isometric exercise on the normal skeletal muscles, which is supposed to trigger a physiological, i.e., functional non-pathological process could improve angiogenesis in remote ischemic areas (6). Lin et al. tested isometric handgrip exercise-induced PIT in 74 patients with coronary artery disease and found that the myocardial perfusion and left ventricular ejection fraction were significantly improved in PIT group. They concluded that PIT may promote remote collateral recruitment and growth (9). To the best of our knowledge, however, the effectiveness and mechanisms of PIT on patients with stroke have not been explored. Therefore, this study is aiming to observe PIT-induced improvement in the remote cerebral ischemia region and to investigate its potential mechanisms.

## Materials and Methods

### Subjects

Twenty patients with acute cerebral infarction were enrolled between April 2013 and March 2015 based on the following criteria: (i) first-time cerebral infarction within 7 days; (ii) aged 18–75 years; (iii) no obvious aphasia or severe cognitive dysfunction; (iv) Fugl–Meyer score ≤75; (v) with stable vital signs; and (vi) signed informed consent form. Exclusion criteria are as follows: (i) combined with brain parenchyma or subarachnoid hemorrhage; (ii) with severe clinical complications, i.e., liver or kidney disease, cardiac arrest or myocardial infarction, severe pneumonia or obstructive pulmonary disease, and refractory hypertension or diabetes; and (iii) with severe cognitive dysfunction. Eligible patients were randomly assigned either into PIT group (*n* = 10) or Control group (*n* = 10). This study was approved by the Ethics Committee of the First Affiliated Hospital of Nanjing Medical University, and all the subjects signed the written informed consent.

### Training Protocol

All the patients received 4 weeks of routine rehabilitation therapy, while patients in the PIT group received additional PIT sections during the treatment period.

#### Routine Rehabilitation Training Program

Patients either in the PIT group or in the Control group went through the routine rehabilitation training program. The routine rehabilitation training program included physical therapy and neuromuscular electrical stimulation. Physical therapy including bed activities, balance training and transfer, and locomotion were used according to patients’ condition for 1 h a day. Neuromuscular electrical stimulation was also usually used in the training. Neuromuscular electrical stimulation was applied on the supraspinatus and deltoids to reduce shoulder subluxation. Wrist extensor was stimulated to launch hand opening. Anterior tibial muscle was stimulated to assist ankle dorsiflexion. The 4.5″ × 6.0″ electrodes were positioned to the muscle with the pulse frequency of 35 Hz and the duration of 20 min. The strength of stimulation changed from 20 to 40 mA according to the condition of patients. The routine rehabilitation training program was used once a day, 5 days a week, for 4 weeks.

#### PIT Program

In addition to the routine rehabilitation training session, patients in the PIT groups also received PIT. PIT was induced by maximal voluntary isometric contraction (MVIC) with handgrip. Patients performed MVIC with a dynamometer for 1 min, followed by 1 min of rest, repeated 10 times a session, 4 sessions a day, and 5 days a week; the same protocol was used on the other extremity. Patients were instructed to maintain normal breathing in order to avoid suffocation during the training.

### Functional Assessment

A well-trained therapist with at least 5 years’ experience was assigned to conduct the assessment. The therapist was blinded to the experimental assignments and treatment. The instructions of all the assessments were fully followed. Assessments include the Fugl–Meyer Assessment (FMA) for motor impairment, the Modified Barthel Index (MBI) for activity of daily living, and the short-form 36-item health survey questionnaire (SF-36) for quality of live. The items and instructions of the above assessments can be found elsewhere (10–12). The scores were recorded according to the results of body check, physical examination, and interlocution.

### Collateral Formation in Brain Ischemic Area by MRI

Conventional MRI T1WI and T2WI series, diffusion-weighted imaging, and dynamic susceptibility contrast enhanced MR perfusion-weighted imaging (DSC-PWI) were undergone for all patients before and after training by using Siemens Verio 3.0 T whole body MR scanner. PWI data were transmitted to Siemens Syngo workstation for processing; the perfusion parameter maps of the regional cerebral blood flow (CBF) were automatically processed. Five pixels (about 0.45 cm^2^) low perfusion area within 1 cm around infarct area were selected as regions of interest (ROIs); the CBF was calculated three times for each side on ROIs covering the low perfusion area and the contralateral mirror area. The collateral formation in ischemia area was described by the CBF ratios; higher ratio indicates better outcome.

### Plasma Measurement of Vascular Endothelial Growth Factor (VEGF)

At the endpoint, serum levels of VEGF were determined using ELISA assays (R&D Systems, USA). Optical density of each well was determined using a microplate reader (Stat Fax-2100, Awareness Corp., USA) at 450 nm. The optical density of each sample was reported as the mean optical density at 450 nm. The obtained values were converted to VEGF levels (picograms per milliliter) through standard formulas.

### Plasma Measurement of Endothelial Progenitor Cells (EPCs)

The number of EPCs was tested by fluorescence-activated cell sorting (BD LSR II with FACS Diva Software, BD Biosciences, USA). Two hundred microliters of whole blood was transferred to polystyrene tubes for testing with the following antibodies added to the samples: APC-conjugated anti-human CD45, FITC-conjugated anti-human CD34 (BD Biosciences, USA), and PE-conjugated anti-human KDR/VEGF-2 (BD Biosciences, USA). The experimental protocol can be found elsewhere (13).

### Statistical Analysis

General clinical data including age, duration of disease, and BMI were compared with independent sample *t*-test, while dichotomous variables such as gender, affected side, history of hypertension, diabetes, hyperlipidemia, smoking, and drinking between groups were compared with chi-square analysis under Yates’s correction. Data of FMA, MBI, and SF-36 at baseline, endpoint, and its corresponding changes were compared with independent sample *t*-test. As the data, in terms of CBF and concentration of VEGF and EPCs, were not normally distributed, non-parametric method (Wilcoxon rank sum test) was adopted, and the results were shown with Box-and-whisker plot. The potential relationships between FMA and CBF, CBF and VEGF, and VEGF and EPCs were predicted with Spearman correlation analysis. *p* < 0.05 was considered as statistical significant.

## Results

### Demographic and Clinical Data

Demographic and clinical data are summarized in Table 1. No significant difference was detected between groups.

**Table 1:** **Demographic and clinical data**.

	PIT group (*n* = 10)	Control group (*n* = 10)	*p*-Value
Age, mean (SD)	59.00 (10.52)	66.40 (8.95)	0.11
Male, *n* (%)	6 (60)	8 (80)	0.63
Duration of disease, mean (SD)	4.40 (0.97)	3.90 (0.88)	0.24
Left side affected, *n* (%)	6 (60)	5 (50)	1.00
**Risk factors**			
BMI, mean (SD)	23.46 (1.37)	23.15 (1.60)	0.65
Hypertension, *n* (%)	9 (90)	9 (90)	1.00
Diabetes, *n* (%)	6 (60)	6 (60)	1.00
Hyperlipidemia, *n* (%)	8 (80)	7 (70)	1.00
Smoking history, *n* (%)	3 (30)	4 (40)	1.00
Drinking history, *n* (%)	4 (40)	3 (30)	1.00

*PIT, physiological ischemic training; BMI, body mass index*.

### Comparisons of Functional Assessments

After 4 weeks of training, the results of FMA, MBI, and SF-36 were significantly higher (*p* < 0.01) than that at baseline in both groups. No significant difference in terms of the results of SF-36, MBI, and FMA were detected between groups at the baseline. Compared to the Control group, the FMA and SF-36 scores in PIT group were significantly higher than that of Control group at the endpoint (*p* < 0.05), while for the value of MBI, no significant difference was detected (Table 2).

**Table 2:** **Results of FMA, MBI, and SF-36 at baseline and endpoint**.

Group	FMA		MBI		SF-36	
	Baseline	Endpoint	Baseline	Endpoint	Baseline	Endpoint
Control group	29.90 ± 13.73	62.90 ± 16.02^a^	26.40 ± 5.28	58.60 ± 14.83^a^	25.00 ± 2.82	37.28 ± 5.70^a^
PIT group	31.80 ± 12.01	66.30 ± 11.98^a^^,^^b^	33.80 ± 17.31	62.90 ± 16.76^a^	25.57 ± 7.19	44.10 ± 7.66^a^^,^^b^

*^a^Compared with pre-training, p < 0.01*.

*^b^Compared with Control group, p < 0.05*.

*FMA, Fugl–Meyer Assessment; MBI, Modified Barthel Index; SF-36, the short-form 36-item health survey questionnaire; PIT, physiological ischemic training*.

### Comparisons of Cerebral Collateral Formation in Two Experimental Groups

**Figure 1:**
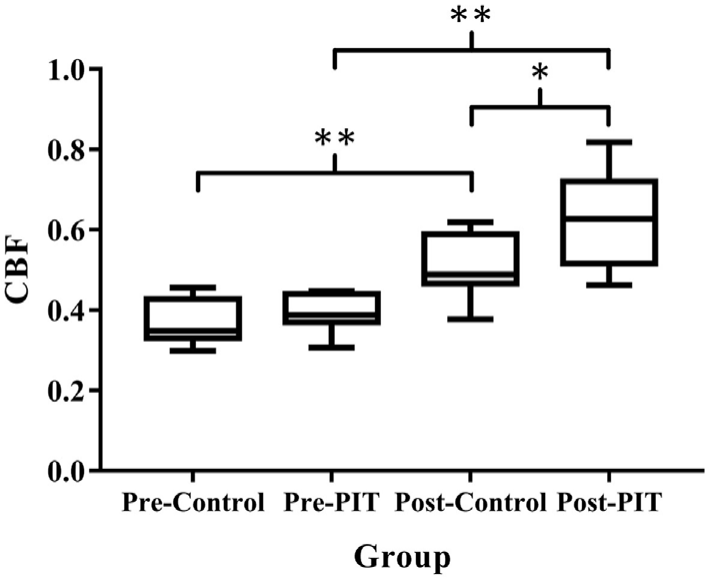
**Comparison of cerebral collateral formation in two experimental groups**. CBF, cerebral blood flow; PIT, physiological ischemic training. Pre-Control indicates data collected from Control group before training; Pre-PIT indicates data collected from PIT group before training; Post-Control indicates data collected from Control group after training; and Post-PIT indicates data collected from PIT group after training. *p < 0.05; **p < 0.01.

As shown in Figure 1, the values of CBF at the endpoint were significantly higher (*p* < 0.01) in both groups than that at the baseline. No significant difference was detected between groups before PIT (*p* > 0.05). As compared to Control group, the value of CBF in PIT group was significantly higher than that of the Control group after PIT (*p* < 0.05).

### Comparisons of Plasma VEGF Level in Two Experimental Groups

**Figure 2:**
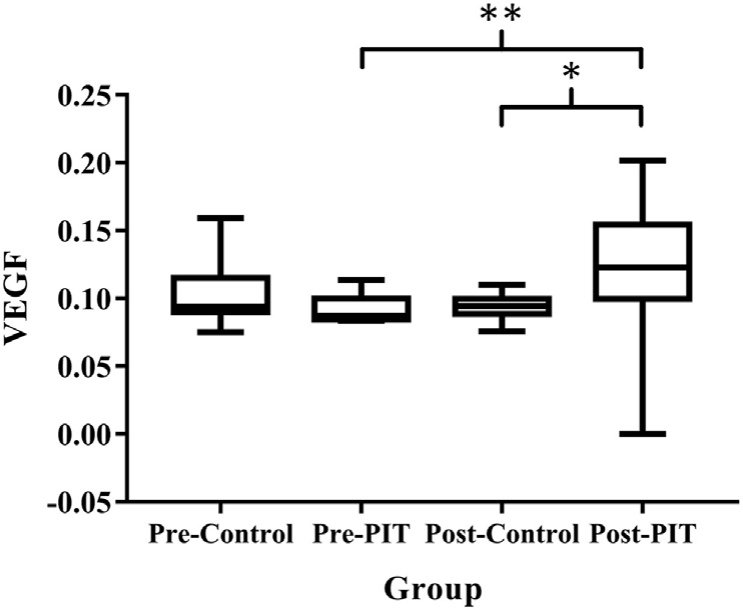
**Comparisons of plasma VEGF level in two experimental groups**. VEGF, vascular endothelial growth factor; PIT, physiological ischemic training. Pre-Control indicates data collected from Control group before training; Pre-PIT indicates data collected from PIT group before training; Post-Control indicates data collected from Control group after training; and Post-PIT indicates data collected from PIT group after training. *p < 0.05; **p < 0.01.

As compared to baseline data, plasma VEGF level in PIT group was significantly higher (*p* < 0.01) at the endpoint, while no significant difference was detected in Control group (*p* > 0.05). For intergroup comparison, plasma VEGF level in the PIT group was significantly higher than that of the Control group after 4 weeks of training (*p* < 0.05) (Figure 2).

### Comparison of Plasma EPCs Number in Two Experimental Groups

**Figure 3:**
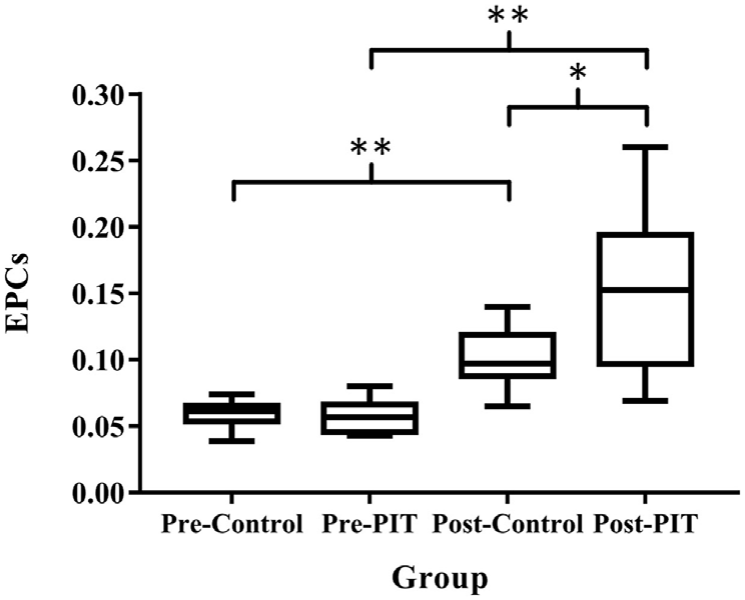
**Comparison of plasma EPCs number in two experimental groups**. EPCs, endothelial progenitor cells; PIT, physiological ischemic training. Pre-Control indicates data collected from Control group before training; Pre-PIT indicates data collected from PIT group before training; Post-Control indicates data collected from Control group after training; and Post-PIT indicates data collected from PIT group after training. *p < 0.05; **p < 0.01.

**Figure 4:**
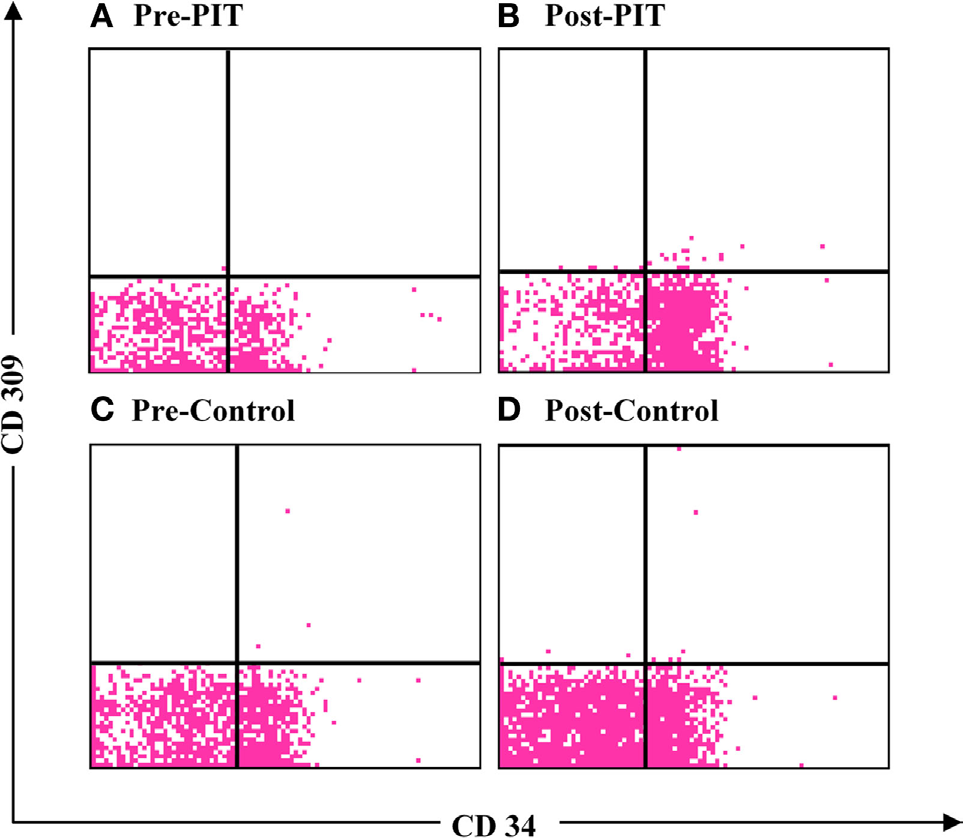
**Flow cytometry of endothelial progenitor cells (EPCs)**. **(A)** Flow cytometry of EPCs in physiological ischemic training (PIT) group at baseline; **(B)** flow cytometry of EPCs in PIT group at endpoint; **(C)** flow cytometry of EPCs in Control group at baseline; and **(D)** flow cytometry of EPCs in Control group at endpoint.

After training, the EPCs number were significantly higher (*p* < 0.01) in both groups than that of baseline. No significant difference was detected in EPCs number between groups before training (*p* > 0.05). As compared to the Control group, the EPCs number in the PIT group was significantly higher than that of the Control group after training (*p* < 0.05) (Figures 3 and 4).

### Correlation between FMA and CBF

**Figure 5:**
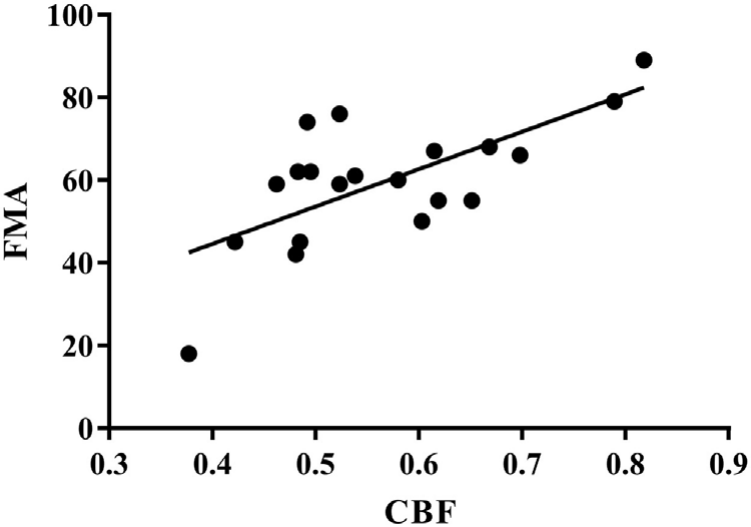
**Correlation between FMA and CBF**. *r* = 0.686, *p* < 0.01. FMA, Fugl–Meyer Assessment; CBF, cerebral blood flow.

As shown in Figure 5, FMA score was positively correlated with CBF (*r* = 0.686, *p* < 0.01).

### Correlation between CBF and Plasma VEGF

**Figure 6:**
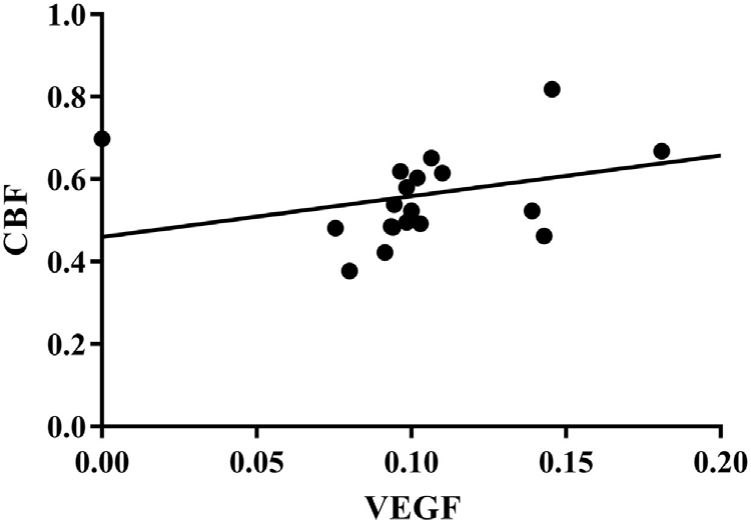
**Correlation between CBF and plasma VEGF**. *r* = 0.675, *p* < 0.01. CBF, cerebral blood flow; VEGF, vascular endothelial growth factor.

As shown in Figure 6, CBF was positively correlated with plasma VEGF (*r* = 0.675, *p* < 0.01).

### Correlation between Plasma EPCs and VEGF

**Figure 7:**
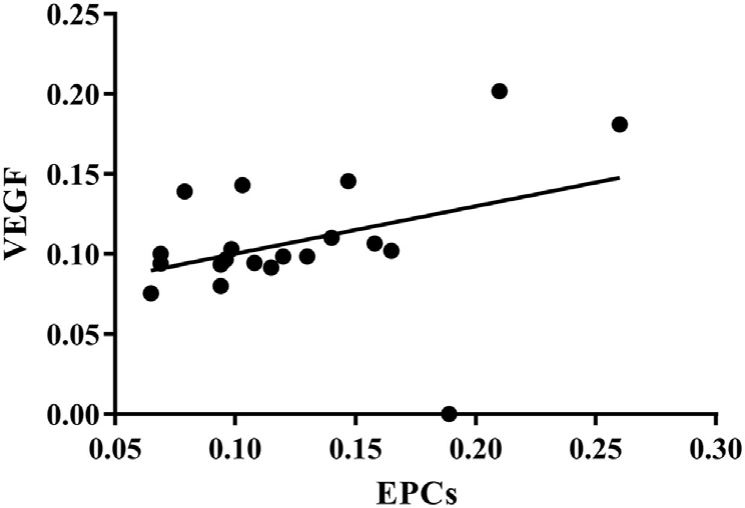
**Correlation between plasma EPCs and VEGF**. *r* = 0.722, *p* < 0.01. EPCs, endothelial progenitor cells; VEGF, vascular endothelial growth factor.

As shown in Figure 7, plasma EPCs number was positively correlated with VEGF level (*r* = 0.722, *p* < 0.01).

## Discussion

In the current study, we found that PIT-induced remote cerebral collateral formation was helpful to improve the functions of patients with acute cerebral infarction. Specifically, PIT can promote the post-ischemic angiogenesis in the brain through increased VEGF and EPCs expression and in turn improve the patients’ movement function and quality of life.

### Effectiveness of PIT on Cerebral Angiogenesis and Functional Recovery in Patients with Acute Cerebral Infarction

The results of the current study showed that after 4 weeks of PIT, the CBF in the PIT group was significantly higher than that of the Control group, thereby indicating that PIT could facilitate the remote brain angiogenesis. For functional recovery in patients with stroke, angiogenetic vessels can provide neurotrophic support to newly generated neurons (14); in addition, neuroblasts have been found to be concentrated around blood vessels following stroke (3). Therefore, neurogenesis and angiogenesis might be mechanistically linked and be able to facilitate the functional recovery of patients with acute cerebral infarction.

Previous animal study showed that three cycles of 15-min occasion/15-min release of left-hind femoral artery after stroke can reduce infarction and ameliorate the outcome of the behavioral test (15). For studies on human, a randomized controlled trial reported that prehospital brief bouts of remote limb ischemia might have immediate neuroprotection for acute cerebral infarction by reduced tissue risk of infarction (16). However, clinical evidence was still limited for non-invasive treatment on the functional recovery of stroke patients. The results of the current study suggested that PIT may promote the recovery of motor function by promoting the formation of collateral circulation in the ischemic area of the brain.

### Mechanism of PIT on Brain Angiogenesis and Functional Recovery

Vascular endothelial growth factor is the major angiogenic factor, which can be induced by exercise, ischemia, hypoxia, and vascular injury (17). Previous study conducted by our research team showed that PIT can increase the expression of VEGF, thereby resulting in accelerating revascularization and coronary blood flow recovery (8). Lin et al. also reported that isometric handgrip-induced PIT could increase the expression of VEGF and the coronary collateral flow in patients with coronary heart disease; the increased VEGF may in turn contribute to collateral angiogenesis in the remote ischemia heart region (9). Sandri et al. observed elevated VEGF level after a 15-min suprasystolic occlusion of one lower extremity in healthy volunteers (18). It is reported that the expression of VEGF significantly increased in the majority of patients after acute stroke. Highest expression occurred at day 7, and it remained significantly elevated 14 days after stroke (19). Our results showed that after 4 weeks of PIT, plasma VEGF level increased significantly (*p* < 0.05) as compared to the Control group, and it was showed that the CBF and plasma VEGF level were positively correlated (*p* < 0.01). Combined with the results from previous studies, it was suggested that the possible mechanism of PIT promoting the recovery of motor function is to increase the VEGF expression and, in turn, facilitate the formation of collateral in cerebral ischemic region in patients with stroke.

Circulating EPCs can home to the site of neovascularization and differentiate into endothelial cells, which can contribute to angiogenesis. Animal studies demonstrated that bone marrow-derived EPCs participate in cerebral neovascularization after focal cerebral ischemia (20). The increased circulating EPCs has been reported to be associated with improved functional outcome and reduced infarction growth in patients with acute ischemic stroke (21, 22).

As EPCs play a critical role in the angiogenesis and functional recovery after brain infarction, it is important to develop an innovative treatment for increasing the endogenous EPCs. Sandri et al. used a maximal treadmill test to induce limb ischemia in patients with peripheral arterial occlusive disease and found that EPCs level increased significantly (18). Lee et al. also observed strong association between increased EPCs level and functional improvement in patients with cerebral infarction (23). Our previous studies showed that isometric exercise-induced PIT can increase circulation EPCs number and in turn facilitate angiogenesis in remote ischemic myocardium. In the current study, the results showed that EPCs number in the PIT group was significantly higher than that of the Control group after training (*p* < 0.05), thereby suggesting that PIT can also increase the EPCs recruitment in stroke patients.

Some studies found that a short episode of myocardial ischemia in CHD patients was sufficient to induce the increase of the circulation EPCs and plasma VEGF (24). Here, in our study, the results showed that PIT can increase the expression of EPCs and VEGF. A linear relationship between EPCs and VEGF was detected; the similar result was also found between VEGF and CBF. These results indicated that PIT may increase the recruitment of EPCs and VEGF, which in turn facilitate the angiogenesis in the cerebral ischemic area.

The major limitation of the current study was the small sample size; caution should be taken when the findings were applied to the specific patients with ischemic stroke, and it may be fixed with the enrollment of more patients in the near future.

In summary, PIT may be effective in increasing the expression of VEGF and recruitment of EPCs, which in turn promote the formation of brain collateral circulation. The positive correlations may demonstrate a potential association between biological and functional parameters, and PIT may be able to improve the motor function, activity of daily living, and quality of life in patients with stroke.

## Author Contributions

All the authors designed the study, performed the study, wrote the manuscript, and read and approved the final version of the paper.

## Conflict of Interest Statement

The authors declare that the research was conducted in the absence of any commercial or financial relationships that could be construed as a potential conflict of interest.
